# Pathological discrepancy between colposcopic directed cervical biopsy and conisation results: A five years experience of a single center in Turkey

**DOI:** 10.12669/pjms.35.6.408

**Published:** 2019

**Authors:** Huseyin Aydogmus, Serhat Sen, Serpil Aydogmus

**Affiliations:** 1 Huseyin Aydogmus, M.D. Izmir Katip Celebi University Atatürk Research and Training Hospital, Department of Gynecology and Obstetrics, Karabaglar, Izmir, Turkey; 2 Serhat Sen, M.D. Izmir Katip Celebi University Atatürk Research and Training Hospital, Department of Gynecology and Obstetrics, Karabaglar, Izmir, Turkey; 3 Serpil Aydogmus, Associate Professor, Izmir Katip Celebi University Scholl of Medicine, Department of Gynecology and Obstetrics, Karabaglar, Izmir, Turkey

**Keywords:** Abnormal smear, Colposcopy, Conisation

## Abstract

**Objective:**

To analyze the clinical outcomes and treatment strategies of patients who underwent conisation in a tertiary hospital clinic

**Methods:**

The study was designed as a retrospective cohort study. Retrospective data’s of 176 patients who underwent conisation due to detection of dysplasia via colposcopic biopsy or cytology-histology discrepancy between 2012 and 2017 were collected. Colposcopy guided biopsies were performed according to HPV positivity and/or abnormal smear results in Izmir Katip Celebi University Ataturk Research and Training Hospital.

**Results:**

Indications for colposcopic biopsies were HPV positivity in 51 patients (29.1%), abnormal smear results in 125 patients (70.9 %). Distribution of abnormal smear results were ASCUS, ASC-H, LSIL, HSIL in 6 (4.8 %), 21 (16.8 %), 24 (19.2%), 74 (59.2%) patients respectively. According to biopsy results, 8 patients (4.4 %) showed no dysplasia where two (1.1%) and 162 (91.5 %) patients were with LSIL and HSIL respectively. Only two were diagnosed with in situ carcinoma. Among 162 patients with HSIL in colposcopic biopsy, 45 showed no dysplasia where four were diagnosed with invasive carcinoma.

**Conclusions:**

To detect high grade cervical lesions colposcopy guided biopsy is gold standard. Although cone biopsy should be performed related to severity of dysplasia in order to sustain the diagnosis and treatment. Contradictory results between colposcopic and cone biopsies should be considered during decision-making process.

## INTRODUCTION

Cervical cancer is second most common cause of death in women due to malignancy after breast cancer globally.^1^ On the other hand, cervical cancer most commonly develops over the course of several years, hence early detection and treatment of cervical dysplasia is critical in preventing the progression to cancer and improving patient outcome. Since widespread use of screening programmes, mortality due to cervical cancer has reduced 14 times in United States.^2^

American Society of Cancer has included HPV testing in the guideline of cervical cancer screening.^3^ Among the population who entered screening programme, five percent needs to have further investigation for positive results.^4^

Colposcopy is gold standard method for evaluating a normal smear and positive high risk HPV results.^5^ Although the most common reason for referral of women for colposcopy is abnormal cervical cytology, clinically suspicious cervix, evaluation of treatment in cervical dysplasia, postcoital bleeding, presence of external genital warts are the other indications for the procedure. To sustain definitive diagnosis and management at the same time, conisation stands for a less invasive and first step procedure.^6^ In this study, we aimed to obtain effective use of colposcopy and further implications leading to conisation or conservative treatment according to our previous practice results.

## METHODS

The datas were scanned from electronic database of our tertiary hospital. Code of colposcopy (620.240) was obtained for data extraction from Turkish Ministry of Health’s Index list of Interventional Procedures. Among those particular individuals, 176 patients who underwent conisation were included in the study. All patients were followed up for at least one year regularly in our clinic.

Abnormal pap-smear was defined as one of the followings, atypical squamous cells of undetermined significance (ASCUS), atypical glandular cells of undetermined significance (AGUS), low-grade squamous intraepithelial lesion (LSIL), high-grade squamous intraepithelial lesion (HSIL) and atypical squamous cells, cannot exclude HSIL (ASC-H).

Diagnostic and treatment procedures were performed by separate clinicians via MDS 3300 colposcop with 2000000 pixels of visual quality, auto focusing, 20 times optical 10 times digitally zooming. Acetowhiteness, punctuation, mosaic pattern, atypical vessels were accepted a normal finding and indicated biopsy (or biopsies for multiple lesions) if detected. Endocervical curettage was performed if necessary. Patients with HSIL underwent excisional procedures. Loop electrosurgical procedures (LEEP) or cold knife conisation was performed according to clinician’s decision.

Demographic data’s (age, parity, menapause status), pap test results and HPV type (if exists), colposcopic and cone biopsy results were recorded to SPSS version 20. Nominal variables were defined as case number and percentage, descriptive variables were calculated as mean ± standard deviation.

## RESULTS

There were one hundred seventy six participants whose mean age was 44.43 ± 11.21, 8 cases (4.5%) were nulliparous, whereas 43 (22.5%) and 125 (73%) were primiparous and multiparous respectively. Median value of parity was 2 (0-10) and 41 (23.5%) cases were postmenapausal.

Indications of colposcopy were divided into HPV-DNA positivity for 51 (29.1%) and abnormal pap test result for 125 (70.1%) cases. Abnormal smear result distribution was, 6 ASCUS (4.8%), 21 ASC-H (16.8%), 24 LSIL (19.2%), 74 HSIL (59.2%). Table-I. Median number of biopsies per person was 3 (1-7). Colposcopic biopsies showed no dysplasia in 8 patients (4.4%) whereas CIN 1 in 2 (1.1%), CIN 2 and CIN 3 in 162 (91.5%), in situ carcinoma in 4 (2.2%) patients.

**Table I:** Abnormal smear result distribution.

Smear	N (176)	No dysplasia	CIN 1	CIN 2,3	Insitu carcinoma	Squamous cancer
ASC-US	6 (%3.4)	4 (%2.2)	1 (%0.55)	1 (%0.55)	-	-
ASC-H	21 (%11.9)	-	-	21(%100)	-	-
AGUS	0	-	-	-	-	-
LSIL	24 (%13.6)	2(%8)	-	21(%88)	1(%4)	-
HSIL	74 (%42)	2(%3)	-	69(%93)	3(%4)	-
HPV +, normal	51 (%29.1)	-	1(%2)	50(%98)		

Six patients whom had colposcopic biopsies due to ASCUS, 4 showed no dysplasia, CIN-1 and CIN-2 was found in the rest, respectively. All cases with HPV positivity without smear test, showed dysplasia in colposcopic findings. Accordingly, except one patient with CIN 1 all patients were diagnosed with CIN 2 or CIN 3 (98.1%). Correlation between colposcopic and cone biopsy results are shown in Table-II.

**Table II:** Correlation between colposcopic and cone biopsy results.

Hystopathology	Colposcopic biopsy	Conisation
No lesion	2(%1.3)	45(%25)
CIN 1	3(%1.7)	6(%5)
CIN 2-3	167(%94.8)	117(%66)
Carcinoma insitu	4(%2.2)	4(%2)
Invasive carcinoma	-	4(%2)

Total	176	176

We found no dysplasia in 45 patients (25.5%) after conisation. Final hystology was reported CIN one in six cases (3.4%) and CIN2 or three in 117 (66.4%). Among 24 patients with LSIL in pap-test, 21 cases were upgraded. On the contrary, 43 of 117 cases with HSIL in colposcopic biopsy showed absence of dysplasia after conisation.

## DISCUSSION

Although cervical cancer screening has been established, discrepancies or lack of agreement between abnormal cervical cytological and subsequent histological findings are not uncommon and present a unique clinical challenge. PAP smear test has low sensitivity, especially in certain centers which have reported 30% sensitivity rates.^3^,^7^ Therefore further investigation is warranted. Colposcopy guided biopsy is still the most effective method even though it has disadvantages such as cost, time and accuracy depending on specific education.

ASCUS is the most common abnormal finding in smear test. Incidence of ASCUS was reported 1.76% in Turkey.^9^ Recent studies suggests HPV testing in the management of ASCUS rather than colposcopy.^10^ Our findings confirmed this algorithm. We have found that all patients with ASC-H cytology had to HSIL in colposcopic biopsy. Thus we suggest ASC-H has to be carefully evaluated through colposcopy as mentioned in the literature.

Surprisingly from 24 patients with LSIL cytology, 21 was upgraded to HSIL in colposcopic biopsy. In contrast, among 117 patients with HSIL in colposcopic biopsy, 43 showed no dysplasia. This may be related with interobserver bias between two pathologists or the time interval between procedures. Studies have reported discrepancy rates between 10-33 % that no dysplasia or less severe lesion could be diagnosed in conisation after colposcopy. Duesing et al. have suggested that difference between two histologies can be explained by total removal of small sized lesions during biopsy, regression via triggered inflammatory reaction or inadequate conisation.^11^ In this context our 36% discrepancy between colposcopy and conisation is higher than reported in earlier studies. As a result, to evaluate cytological findings with severe dysplasia, colposcopy guided biopsy is crucial. However in most cases, adequate conisation is warranted to obtain definitive diagnosis and treatment.

The discrepancy of concordance between colposcopically directed punch biopsy and subsequent histopathological conization finding is common and present a unique clinical challenge. Limited accuracy of colposcopically directed punch biopsy and inconsistency of pathology with LEEP conization remain an important clinical problem. Biopsy results that underestimate the CIN grade (biopsy underestimation) may have serious implications if conization was not performed. Patients age, number of deliveries, HPV type, number of biopsies and experience of pathologists should effect the correlation between conisation and colposcopic biopsy results and may lead to underestimation or overestimation of clinical findings. The number of our cohort is a major limitation to make logistic regression analysis.

### Limitations of the study

First, procedures and pathological evaluations were performed by different clinicians. This may cause interobserver bias. Secondly, our sample size is relatively small. Nevertheless we believe our findings enlights pitfalls and possible improvements to conducted prospective trials such as our groups ongoing study.

## CONCLUSION

Even PAP smear and HPV-DNA testing are highly effective methods on cervical cancer screening, they still have low sensitivity and predictive values. Thus, colposcopy has a key role on determining abnormal screening results and high risk HPV infection. Since there is notable difference of opinion between colposcopy and cone biopsy results further development and standartisation in colposcopic management is necessary.

### Authors’ Contribution:

**HA:** Study design and concept, data analyses, data interpretation, preparation of manuscript.

**SS:** Study design and concept, data collection, drafting the paper.

**SA:** Study design, literature search, drafting the manuscript.
